# A Fatal Case of Clostridium septicum Mycotic Aortic Aneurysm: The Inadequacy of Thoracic Endovascular Aortic Repair in the Absence of Open Surgical Repair

**DOI:** 10.7759/cureus.110833

**Published:** 2026-06-14

**Authors:** Colton H Connor, Niraj R Rama, Daisy Sangroula, Abindra Sigdel

**Affiliations:** 1 Department of Surgery, Division of Vascular Surgery, School of Medicine, University of Louisville, Louisville, USA; 2 Department of Anesthesiology and Perioperative Medicine, School of Medicine, University of Louisville, Louisville, USA

**Keywords:** aortitis, clostridium septicum, mycotic aneurysm, open aortic repair, tevar

## Abstract

*Clostridium septicum* is a rare, highly virulent Gram-positive, spore-forming anaerobic bacterium that preferentially colonizes hypoxic and necrotic tissue and is associated with underlying malignancy, particularly gastrointestinal cancers. *C. septicum*-associated mycotic aortic aneurysm is an uncommon but highly lethal complication. We report a case of a 64-year-old man with a history of squamous cell carcinoma of the head and neck who presented with central chest pain and was found to have a 9.2 × 7.5 × 6.2 cm necrotic mediastinal mass inseparable from the aortic arch, *C. septicum* bacteremia, and a 6 cm mycotic aneurysm of the distal aortic arch. Open repair was declined by cardiothoracic surgery due to the patient's prohibitive medical condition, and thoracic endovascular aortic repair (TEVAR) was performed as the only available surgical option. The patient initially recovered but returned two months later with hematemesis from progressive aortic degeneration and aortoenteric fistula despite endograft revision, and he died shortly thereafter. This case expands the literature on *C. septicum* aortitis by documenting direct contiguous extension from an adjacent necrotic mass as a viable mechanism of aortic infection and demonstrates the inadequacy of TEVAR as a standalone therapy when open surgical repair is not feasible. Early recognition, definitive open aortic repair when possible, and a multidisciplinary approach integrating vascular surgery, infectious disease, and oncology are essential to optimizing outcomes in this rare but lethal disease.

## Introduction

*Clostridium septicum* is a rare but highly virulent Gram-positive, spore-forming anaerobic bacterium seen in 1.3% of infections of the Clostridium genus [1-5]. *C. septicum* is known to cause life-threatening infections, including gas gangrene and myonecrosis due to alpha toxin production and has been linked to malignancy in 82.5% of cases [3,5,6]. Due to its anaerobic nature, the bacterium has a high potential to multiply in hypoxic and necrotic tissue, leading to translocation through the bowel mucosa and systemic vascular destruction, possibly resulting in sepsis [2]. Notably, *C. septicum* has been shown to cause mycotic aortic aneurysms secondary to aortitis, although fewer than 60 reported cases of this occurrence have been documented [3,7]. Mycotic aneurysms result from local dilation and compromise of the aortic tunica intima from bacterial activity or direct infection of adjacent vascular structures [8]. Without prompt surgical intervention, these infected aneurysms carry a high risk of rupture, resulting in catastrophic hemorrhage. Without surgical intervention, the prognosis of *C. septicum*-associated aneurysms approaches a six-month 100% mortality rate, with most of the literature demonstrating near 100% mortality at one year [2-4,6,7,9]. Open aortic repair (OAR) itself carries a 90-day mortality of approximately 30%, with still very few patients undergoing invasive surgical treatment surviving longer than a year [2,3]. The use of antibiotics represents an option to mitigate the caustic effects of *C. septicum* but has been deemed an adjunct approach to treatment, with definitive antibiotic regimens being limited to a palliative option [2-4]. Thoracic endovascular aortic repair (TEVAR) has emerged as an increasingly utilized alternative for thoracic aneurysm repair, given its lower perioperative morbidity compared with open surgical repair. However, TEVAR is often inadequate for *C. septicum* mycotic aneurysms as endovascular grafts provide only a mechanical seal without eliminating the underlying infection, and they do not permit the wide debridement of infected aortic tissue that is fundamental to managing mycotic disease [2,3]. As a consequence, infection may persist or extend along the aorta, leading to loss of endograft seal, ongoing pseudoaneurysm formation, and aortoenteric fistula [3]. Thus, early recognition of this condition is critical to improve prognosis through aggressive surgical and antimicrobial therapy.

## Case presentation

A 64-year-old man with a history of hypertension, hyperlipidemia, stroke (cerebrovascular accident {CVA}), and squamous cell carcinoma of the head and neck presented to the emergency department at an outside hospital with central chest pain and a productive cough. A PET scan performed a week prior at an outside facility for squamous cell carcinoma of the head and neck reportedly revealed a mediastinal mass and a subpleural pulmonary nodule in the right lower lobe. A computed tomography angiography (CTA) of the chest at the outside emergency department showed evidence for the development of a large aneurysm of the distal aortic arch measuring up to 6 cm in diameter, with the possibility of erosion into and communicating with the necrotic mediastinal mass. Airway compression and deviation were also noted, and the patient was transferred to our institution. Cardiothoracic and vascular surgery teams were consulted, and an infectious workup revealed that one of three blood cultures was positive for *Clostridium septicum*.

Upon presentation to our center, the patient had developed hoarseness. A CT of the chest with contrast confirmed a 9.2 × 7.5 × 6.2 cm solid mediastinal mass centered in the left paratracheal and aortopulmonary window region with extension into the subcarinal region and left hilum (Figures 1A, 1B). The mass was inseparable from the aortic arch, descending aorta, esophagus, trachea, left mainstem bronchus, and left main pulmonary artery. With large, irregular aneurysmal degeneration of the aortic arch concerning for mycotic aneurysm, the patient was promptly started on antibiotics, and a surgical workup was initiated. Initially, the cardiothoracic surgery team was consulted for an open excision and repair of the presumed Clostridium-associated mycotic aneurysm. The cardiothoracic surgery team declined open surgical repair, citing the patient’s poor medical condition. At this time, the vascular surgery team offered a thoracic endovascular aortic repair (TEVAR) for the impending rupture of the mycotic aneurysm involving the aortic arch.

**Figure 1:**
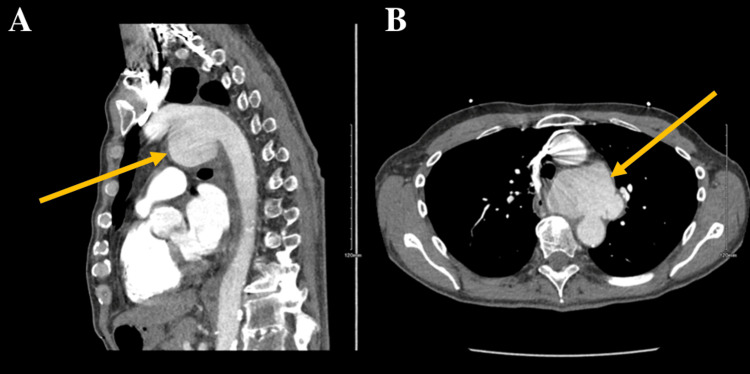
Sagittal (A) and axial (B) contrast-enhanced CT of the mediastinal mass involving the aortic arch. Sagittal (A) and axial (B) contrast-enhanced CT views demonstrating a 9.2 × 7.5 × 6.2 cm solid mediastinal mass (arrows) centered in the left paratracheal and aortopulmonary window region, extending into the subcarinal region and left hilum. The mass is inseparable from the aortic arch, descending aorta, esophagus, trachea, left mainstem bronchus, and left main pulmonary artery.

The procedure was performed under general anesthesia. Using ultrasound guidance, the right common femoral artery was accessed. A wire was advanced into the thoracic aorta, followed by placement of a 22-French sheath in the distal aorta. Pre-deployment aortography demonstrated the large aneurysm of the distal aortic arch (Figure 2A). Following this, the origin of the subclavian artery was cannulated, and a wire was advanced distally into the brachial artery. A 10 mm Amplatzer Vascular Plug II (North Chicago, IL: Abbott Laboratories) was then deployed in the proximal subclavian artery. A Gore C-TAG endograft (Flagstaff, AZ: W. L. Gore & Associates, Inc.) measuring 31 mm × 10 cm was subsequently deployed in the proximal descending thoracic aorta. A proximal extension endograft with another C-TAG measuring 34 mm × 10 cm was then placed adjacent to the origin of the left common carotid artery, covering the left subclavian artery. Post-deployment angiography confirmed the patency of the innominate and carotid arteries and adequate coverage of the left subclavian artery (Figure 2B). After coverage and embolization of the subclavian artery, the patient had a mean radial arterial line pressure reading of 70-80 mmHg; therefore, no carotid-to-subclavian bypass was performed. However, plans were made for continuous monitoring of the left arm blood pressure and for possible future intervention, in the form of a left carotid-to-subclavian artery bypass, if necessary. The patient tolerated the surgery well and had an uncomplicated recovery.

**Figure 2:**
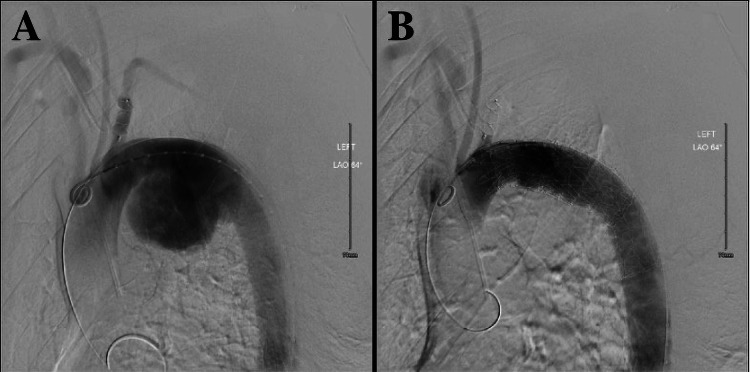
Intra-operative aortography in the left anterior oblique (LAO) projection. Pre-deployment aortogram demonstrating the large aneurysm of the distal aortic arch measuring up to 6 cm in diameter (A). Post-deployment aortogram showing the thoracic endograft and left subclavian artery plug in place with complete exclusion of the aneurysm sac (B).

Post-operatively, an infectious disease specialist was consulted for a long-term antibiotic regimen. The patient was subsequently placed on a continuous infusion of 24,000,000 units of penicillin G every 24 h for six weeks, followed by 500 mg of oral Pen-VK every 8 h for life. One week later, a gallium scan was performed, which demonstrated moderate focal uptake in the midline of the anterior upper abdomen, separate from normal uptake in the transverse colon, raising concern for an abdominal aortic graft infection. Eventually, the patient was discharged home with instructions to continue antibiotic treatment as prescribed. A few weeks after discharge, he presented to a local hospital with persistent gastrointestinal bleeding. Workup revealed a bleeding cecal mass, for which he received a right hemicolectomy in his hometown.

Two months later, the patient presented to the local, outside emergency department with massive hematemesis. Imaging there revealed further degeneration of the aortic arch with loss of seal from the previously placed thoracic endograft. A leak was observed at both the proximal and distal ends of the endograft, indicating extension of the infection into the previously uninvolved portion of the aorta. The findings were concerning for a possible aortoenteric fistula to the distal esophagus, and the patient was immediately transferred to our institution for emergent surgery.

Upon arrival, the patient was hemodynamically unstable and had already been intubated for airway protection. Given the patient’s extremely poor clinical status and complicated pathology, the family was informed of the dreary prognosis, with near-certain mortality from the procedure. The vascular surgery team clearly communicated that no viable endovascular solution was available at this time, as further extension of the endograft was not expected to achieve an adequate seal. The family was advised to strongly consider comfort care measures. The patient’s family, however, insisted on surgical intervention despite the high probability of a poor outcome.

At this point, the patient was transferred to a hybrid endovascular suite. Via femoral arterial access, a thoracic aortic angiography was performed. The aortogram revealed large leaks both proximal and distal to the aortic endograft (Figure 3). Due to the lack of options for proximal extension, only the distal pseudoaneurysm was addressed, with the hope of controlling hematemesis that possibly originated from the aneurysm at the distal edge of the existing endograft. A 31 × 10 mm Gore C-TAG graft was deployed within the existing endograft and distally into the native thoracic aorta, covering the pseudoaneurysm. A repeat aortogram confirmed complete pseudoaneurysm coverage with preserved distal flow. The patient remained unstable throughout the surgery. Ultimately, the patient was transferred to the ICU in critical condition. However, in the hours following the surgery, the patient began to drain bright red blood through the nasogastric tube, followed by hemodynamic collapse. Prompt CPR was initiated but was discontinued a few minutes later when the family members decided to forgo further resuscitation. The patient passed away shortly thereafter.

**Figure 3:**
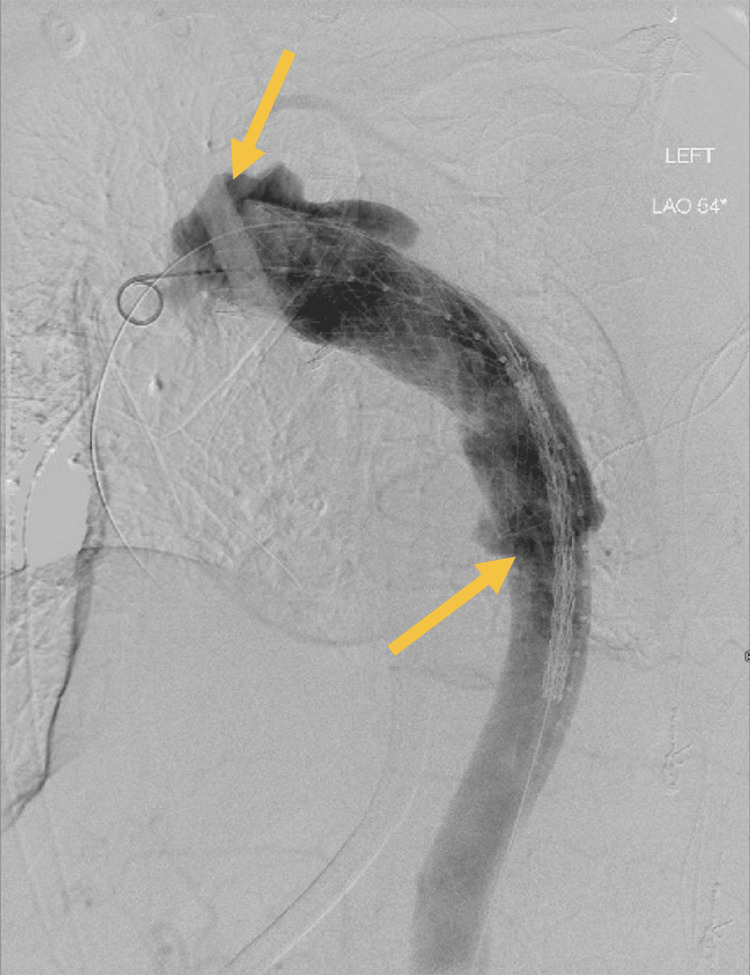
Aortogram under LAO projection revealed proximal loss of seal, aneurysm near the previously covered left subclavian artery (upper arrow), and a large pseudoaneurysm at the distal graft end (lower arrow), indicating rapid mycotic behavior. LAO: left anterior oblique

## Discussion

*Clostridium septicum* is a highly pathogenic anaerobic bacterium that preferentially colonizes regions of relative hypoxia in the body, including malignancies, atherosclerotic plaques, and the gut mucosa [2,3,5,6,9,10]. Malignancies, in particular, are strongly associated with non-traumatic gas gangrene and myonecrosis due to the unique microenvironment created by tumors [6,9]. This process is accelerated by the release of alpha-toxin (hemolysin) and other toxins, including hyaluronidase and lethal necrotizing toxin [4,9,11]. Malignant tissues often outgrow their blood supply, resulting in hypoxic and necrotic areas with reduced immune surveillance, facilitating *C. septicum* colonization. *C. septicum* spores can proliferate under mucosal conditions such as gastrointestinal malignancies, colitis, and diverticulosis, potentially leading to bloodstream infection through intestinal ulcerations [9,12]. This preferential targeting is often associated with gastrointestinal malignancies, such as colorectal and hematologic cancers, highlighting the role of tumor microenvironments in opportunistic infections [1,2,4,6,7,10,13]. Our patient likely had an undiagnosed gastrointestinal malignancy at initial presentation, despite a negative colonoscopy six months prior. Diverticulosis was also present and has been linked as a risk factor for *C. septicum* infection and subsequent bacteremia, although infection in the absence of gastrointestinal malignancy is exceedingly rare [7]. Whether earlier identification of the cecal mass could have altered the clinical course is uncertain. The colonoscopy six months prior to presentation was negative, and the malignancy became clinically apparent only after the development of *C. septicum* bacteremia and TEVAR. Had the cecal malignancy been identified at initial presentation, source control with hemicolectomy and oncologic management could have been pursued alongside vascular intervention, potentially reducing ongoing bacteremic seeding. The established aortic infection and the dominant operative risks posed by the mediastinal mass, however, would not have been substantially altered.

In our patient, the gastrointestinal mucosa likely served as the source of *C. septicum* bacteremia, with the bacteria subsequently seeding the necrotic mediastinal mass. While the more commonly reported mechanism of *C. septicum* aortitis is direct hematogenous seeding of atherosclerotic plaques in the aortic wall, the imaging findings in our case strongly suggest a different route to aortic involvement [4,8,12]. Cross-sectional imaging demonstrated that the necrotic mediastinal mass was inseparable from the aortic arch and descending aorta with concurrent development of a mycotic aneurysm at the same anatomic location, supporting contiguous spread of infection from the mass into the adjacent aortic wall. This case demonstrates that direct extension from a hematogenously seeded necrotic focus represents an underrecognized mechanism of *C. septicum* aortitis, distinct from the more typical route of direct aortic seeding.

In recent years, TEVAR has become an increasingly utilized approach for thoracic aneurysm repair due to its minimally invasive nature and lower morbidity compared to open surgical repair [14]. However, TEVAR may have limited efficacy against *C. septicum* mycotic aneurysms. Endovascular grafts provide a mechanical seal but fail to eliminate the underlying infection, leading to persistent infection, as seen in our patient. Unlike open repair, TEVAR does not permit extensive debridement of infected tissue, a critical step in managing mycotic aneurysms [15]. A successful case of open repair for a *C. septicum* mycotic aneurysm has been reported in the literature. The patient in that case underwent complete resection of the infected aorta and total aortic arch replacement, remaining healthy and asymptomatic two years post-operatively [3]. Few cases of patient survival beyond one year following open aortic repair for *C. septicum *mycotic aneurysm have been documented in the literature. Unfortunately, our patient was not a candidate for open repair given several prohibitive factors. Anatomically, the 9.2 × 7.5 × 6.2 cm necrotic mediastinal mass was inseparable from the aortic arch, descending aorta, esophagus, trachea, left mainstem bronchus, and left main pulmonary artery, obliterating the surgical planes required for total aortic arch replacement. Physiologically, the patient's active head and neck malignancy, airway compromise from mass effect with associated hoarseness, and the systemic burden of *C. septicum* infection compounded the operative risk of a procedure that would have likely required sternotomy, cardiopulmonary bypass with deep hypothermic circulatory arrest, and extensive dissection through an actively infected and tumor-encased operative field. Within these constraints, TEVAR was undertaken as the only available option to address impending rupture. Thus, potential strategies of combination management with TEVAR and antibiotics warrant further investigation for patients unable to undergo open surgery, as open aortic repair yields the best prognosis for patients with a diagnosis of *C. septicum* aortitis [2].

## Conclusions

In conclusion, this case of *Clostridium septicum* aortitis underscores the aggressive and often fatal course of this rare infection. Even with aggressive antibiotic therapy and surgical intervention, prognosis remains poor. Early open aortic repair with extensive debridement followed by prolonged antibiotic therapy appears to offer the best survival outcomes. For patients with prohibitive medical conditions, TEVAR may serve as the only available surgical option or as a temporizing measure prior to definitive open repair. This case additionally illustrates direct extension from an adjacent necrotic mediastinal mass as a viable mechanism of aortic infection and highlights the need for a multidisciplinary approach integrating vascular surgery, infectious disease, and oncology. Given the exceptionally high mortality associated with *C. septicum* aortitis, further investigation into combination strategies, including novel antimicrobial regimens and hybrid surgical approaches, is warranted to improve outcomes in patients for whom open aortic repair is not feasible.

## References

[1] Alimi Y, Sosin M, Borsinger TM, Garrett JR, Salameh JR, Espinel CF (2017). Implications of Clostridium septicum in vascular surgery: a case report and outcomes literature review. Ann Vasc Surg.

[2] Kirchweger P, Wundsam H, Bosse F, Kupferthaler A, Huemer R, Függer R (2022). Prospects of conservative treatment for Clostridium septicum-associated aortitis. J Vasc Surg Cases Innov Tech.

[3] Lehman B, Miller RM, Richter SS (2020). Clostridium septicum-infected aortic aneurysm or graft is a deadly diagnosis. J Vasc Surg.

[4] Seder CW, Kramer M, Long G, Uzieblo MR, Shanley CJ, Bove P (2009). Clostridium septicum aortitis: report of two cases and review of the literature. J Vasc Surg.

[5] Alpern RJ, Dowell Jr VR (1969). Clostridium septicum infections and malignancy. JAMA.

[6] Ito F, Inokuchi R, Matsumoto A (2017). Presence of periaortic gas in Clostridium septicum-infected aortic aneurysm aids in early diagnosis: a case report and systematic review of the literature. J Med Case Rep.

[7] Bahn AJ, Osherov A, Lau DL, Safran BA, Brewer MB (2022). Mycotic aortic aneurysm due to Clostridium septicum and diverticulitis. Ann Vasc Surg Brief Rep Inn.

[8] Raavi L, Garg P, Hussain MW (2022). Mycotic thoracic aortic aneurysm: epidemiology, pathophysiology, diagnosis, and management. Cureus.

[9] Urgiles S, Matos-Casano H, Win KZ, Berardo J, Bhatt U, Shah J (2019). Emphysematous aortitis due to Clostridium septicum in an 89-year-old female with ileus. Case Rep Infect Dis.

[10] Moseley B, Mwirigi NW, Bowen J (2010). Clostridium septicum aortitis and cecal adenocarcinoma. Case Rep Med.

[11] Kennedy CL, Krejany EO, Young LF (2005). The α-toxin of Clostridium septicum is essential for virulence. Mol Microbiol.

[12] Annapureddy N, Agarwal SK, Kanakadandi V (2012). Clostridium septicum aortitis in a patient with extensive atheromatous disease of the aorta. J Infect Chemother.

[13] Srivastava I, Aldape MJ, Bryant AE, Stevens DL (2017). Spontaneous C. septicum gas gangrene: a literature review. Anaerobe.

[14] Nation DA, Wang GJ (2015). TEVAR: endovascular repair of the thoracic aorta. Semin Intervent Radiol.

[15] Tokuda T, Tamaki M, Kitamura H (2019). A mycotic aortic aneurysm treated by thoracic endovascular aneurysm repair. J Surg Case Rep.

